# Multiple Light-Dark Signals Regulate Expression of the DEAD-Box RNA Helicase CrhR in *Synechocystis* PCC 6803

**DOI:** 10.3390/cells11213397

**Published:** 2022-10-27

**Authors:** Sean P. A. Ritter, Logan A. Brand, Shelby L. Vincent, Albert Remus R. Rosana, Allison C. Lewis, Denise S. Whitford, George W. Owttrim

**Affiliations:** 1 Department of Botany, University of British Columbia, 3156-6270 University Blvd., Vancouver, BC V6T 1Z4, Canada; 2 Department of Biological Sciences, University of Alberta, Edmonton, AB T6G 2E9, Canada; 3 Max Planck Institute of Molecular Cell Biology and Genetics, Pfotenhauerstraße 108, 01307 Dresden, Germany

**Keywords:** cold stress, CrhR RNA helicase, light-dark transition, phytochrome, plastoquinone redox poise, post-transcriptional gene regulation

## Abstract

Since oxygenic photosynthesis evolved in the common ancestor of cyanobacteria during the Archean, a range of sensing and response strategies evolved to allow efficient acclimation to the fluctuating light conditions experienced in the diverse environments they inhabit. However, how these regulatory mechanisms are assimilated at the molecular level to coordinate individual gene expression is still being elucidated. Here, we demonstrate that integration of a series of three distinct light signals generate an unexpectedly complex network regulating expression of the sole DEAD-box RNA helicase, CrhR, encoded in *Synechocystis* sp. PCC 6803. The mechanisms function at the transcriptional, translational and post-translation levels, fine-tuning CrhR abundance to permit rapid acclimation to fluctuating light and temperature regimes. CrhR abundance is enhanced 15-fold by low temperature stress. We initially confirmed that the primary mechanism controlling *crhR* transcript accumulation at 20 °C requires a light quantity-driven reduction of the redox poise in the vicinity of the plastoquinone pool. Once transcribed, a specific light quality cue, a red light signal, was required for *crhR* translation, far-red reversal of which indicates a phytochrome-mediated mechanism. Examination of CrhR repression at 30 °C revealed that a redox- and light quality-independent light signal was required to initiate CrhR degradation. The crucial role of light was further revealed by the observation that dark conditions superseded the light signals required to initiate each of these regulatory processes. The findings reveal an unexpected complexity of light-dark sensing and signaling that regulate expression of an individual gene in cyanobacteria, an integrated mechanism of environmental perception not previously reported.

## 1. Introduction

Sensing and the molecular response to fluctuating light conditions are crucial for the survival of photosynthetic organisms. This is especially true for free-living cyanobacteria, many of which are obligate photoautotrophs that obtain all of their energy from light harvesting and carbon skeletons from photosynthetic carbon fixation. Cyanobacteria and higher plants have therefore evolved a range of light sensing systems that regulate gene expression to coordinate the physiological and metabolic responses required for acclimation [1,2,3,4,5,6].

In all photosynthetic organisms, light driven alteration of the redox state of the photosynthetic electron transport chain (ETC) is a known sensor that regulates gene expression. Signal transduction pathways originating from the redox poise of the ETC components plastoquinone (PQ) and ferredoxin (Fd) regulate gene expression in cyanobacteria, algae and higher plants [7,8] and are crucial to integrating energy homeostasis with core metabolism [9] and response to abiotic stress [10,11]. Since reactive oxygen species (ROS) production is oxygen dependent and a natural consequence of light driven electron flow through the ETC in photosynthetic organisms, identification of gene expression regulation by each of these signals is crucial. Barth et al. [12] utilized an interlinked experimental system to evaluate the individual contribution of light, redox, oxygen or ROS regulation using a proteomics approach in *Chlamydomonas reinhardtii*. The authors identified five mechanisms that regulated distinct protein expression profiles in this alga [12]. The diversity of these mechanisms uniquely associated with light regulation, demonstrate the complexity of gene expression regulation downstream of light perception. 

Light quality, quantity and the relative redox poise of the ETC differentially regulate distinct aspects of gene expression and functioning of the photosynthetic machinery. While both photosystems absorb red and blue light, they do so to different extents. Blue light absorption by chlorophyll a (Chl a) creates excess reduction of PSI and a deficiency of electron flow through the ETC at PSII [13]. The resulting imbalance in reduction of the ETC is compensated for by red-light harvesting via phycobilisomes (PBS), primarily associated with PSII, that have negligible absorption in the blue at wavelengths below 495 nm [14]. While red light is also absorbed by Chl a in both photosystems, it generates divergent effects from blue light when supplied at wavelengths around 660 nm. This wavelength is absorbed by allo-phycocyanin in the PBS core, allowing redistribution of photons over both photosystems [13]. Red light is therefore a crucial regulator of gene expression, response to abiotic stress and behaviour in cyanobacteria since it stimulates ETC reduction at PQ [15,16,17,18]. Similar to plant systems, red to far-red photoreversibility is mediated by the photoreceptors Cph1 and Cph2 that differentially regulate gene expression and growth in the model cyanobacterial species, *Synechocystis* sp. PCC 6803 (hereafter *Synechocystis*) [15,19]. Many of the genes exhibiting Cph1/2-mediated red to far-red regulation have also been reported to be regulated by the redox poise of the ETC [19]. It has also been suggested that Cph1/2 effects potentially involve regulation at the post-transcriptional level [6]. Blue light sensing is also important in cyanobacteria as for example, although the mechanism is not known, short daily exposures of blue light are essential for chemoheterotrophic growth in complete darkness, suggesting that while not important as a source of energy, blue light functions as an environmental signal regulating cyanobacterial gene expression and metabolism [20,21]. This differential contribution of light quality to photosynthesis is distinctly different from that observed in algae and higher plants [13].

Expression of *crhR*, encoding the single DEAD-box RNA helicase in *Synechocystis* is regulated by a range of abiotic stresses via their common effect on the redox state of PQ [10,11]. RNA helicases function in all aspects of RNA metabolism through the ATP-dependent rearrangement of RNA secondary structure or ribonucleoprotein interactions [22]. These RNA secondary structure alterations are known to regulate gene expression, frequently in response to abiotic stress, in association with alteration of small-regulatory RNA (sRNA) activity and RNA processing [22,23,24,25,26]. Associated with the redox poise of PQ, temperature shift dramatically affects *crhR* expression, downshift from 30 to 20 °C enhances both transcript and protein abundance by 10–15 fold [11,27] while subsequent upshift to 30 °C induces temperature-dependent, conditional proteolysis of CrhR [28]. These temperature-mediated regulatory pathways involve a complex interconnected network consisting of a number of CrhR-dependent auto-regulatory and CrhR-independent steps [25,27,28]. Thus, evidence has been provided indicating that the light-driven alteration of the PQ redox poise regulates *crhR* expression, coupling gene expression with cellular energy homeostasis and survival at 20 °C in response to environmental conditions that cause over-reduction of the PQ pool [10,11,27,29].

Interestingly, divergent results have been presented regarding the requirement of light for low temperature induction of *crhR* transcript accumulation. We initially reported that *crhR* was not detected in dark incubated cells at 20 °C but rapidly accumulates in the presence of light [10]. Similarly, proteomic analysis indicated that light was required for CrhR protein accumulation [30]. In contrast, data was presented indicating that light was not required for *crhR* transcript accumulation at 22 °C, as transcript was detected in cold treated cells in the dark [17,31]. Overall, these results indicate that light performs multiple roles associated with *crhR* expression. However, the specific aspects of how light regulates *crhR* at each level of gene expression has yet to be investigated.

Here, we expand this analysis by further investigating the contribution of light sensing and signaling to *crhR* expression in *Synechocystis*. The results reveal that three distinct light signals facilitate regulation at multiple stages of gene expression associated with: (1) light-quantity driven alteration of the redox poise of PQ, required for transcript accumulation in response to abiotic stress; (2) a light quality signal where CrhR translation, but not transcript accumulation, is associated with a phytochrome-mediated red-far-red light signal; and (3) a light cue per se, independent of redox poise and light quality, required for the proteolytic repression of CrhR abundance in the absence of stress. Insightfully, these regulatory processes are not observed in dark treated cells, revealing an unexpected complexity of light-dark sensing and signaling associated with CrhR expression in *Synechocystis*.

## 2. Materials and Methods

### 2.1. Strains and Culture Conditions

The glucose tolerant strain of *Synechocystis* sp. PCC 6803 [10] was grown under standard conditions in BG-11 medium at 30 °C under continuous white light at 50 μmol m^−2^ s^−1^ and aerated by shaking at 150 rpm and bubbling with humidified air as described previously [32]. When described, a genomic truncation mutant of the *crhR* ORF, *crhR*_TR_, was grown in the presence of spectinomycin and streptomycin, both at 50 mg/mL [29]. The *crhR*_TR_ strain produces a ~750 nt truncated version of the *crhR* transcript and a 27 kDa polypeptide that is biochemical inactive. Bubbling with exogenous gasses was performed as described for humidified air. A microaerobic environment was provided by bubbling with a 95% N_2_-5% CO_2_ mixture. Photosynthetically active radiation (36 μmol m^−2^ s^−1^) was provided by a bank of warm-white fluorescent lamps and corresponded to low light (LL) conditions. For mid light conditions (ML), warm-white fluorescent lamps provided photosynthetically active radiation at 150 μmol m^−2^ s^−1^. Light quality was altered using Innova Dual Spectrum LED Grow Lights providing red (662 nm) or blue (455 nm) illumination at 30 μmol m^−2^ s^−1^ individually or 40 μmol m^−2^ s^−1^ in combination. Green and far-red illumination was provided as follows: Green (550 nm; MODE Electronics, Burnaby, BC, Canada) at 30 μmol m^−2^ s^−1^ and far-red light (730 nm; ShenZhen HuaKe Light Electronics Co., Ltd., Shenzhen, China). Experiments utilized cultures with chlorophyll *a* (Chl *a*) concentrations ranging from 5–7 µg/mL. Technical replicates involved triplicate samples from the same aliquot from a single culture for qPCR analysis. Biological replicates for western analysis and qPCR were performed on a minimum of two independent cultures grown at least a week apart, with representative data shown. All experiments, except for the antibiotic effects, were also performed over a time course to illustrate both linear responses with respect to time and also reversal of the induction or repression effects.

### 2.2. Stress and Inhibitor Conditions

Unless otherwise stated, illuminated mid-log phase cultures were subjected to the following stress conditions for the indicated times: 20 °C cold stress, medium light (ML) (150 μmol m^−2^ s^−1^). Enhanced CO_2_ levels were provided by bubbling with 5% CO_2_ in either nitrogen or air to provide either microaerobic or aerobic conditions, respectively. The electron transport inhibitors 3-(3,4-dichlorophenyl)-1,1-dimethylurea (DCMU, 2.5 µM) and dibromothymoquinone (DBMIB, 10 µM) were dissolved in DMSO as described previously [10]. DMSO (0.066%) was added to a control culture when ETC inhibitors were used.

### 2.3. Protein Abundance

Wild type CrhR (55 kDa) abundance was determined by Western blot analysis of soluble protein (20 µg) using anti-CrhR antibody (1:5000) (9) and ECL detection (ECL, Bio-Rad Clarity Western ECL Substrate, Mississauga, ON, Canada), as previously described [32]. Stained gels and/or the abundance of Rps1 were used as a protein loading controls using anti-*Escherichia coli* Rps1 (1:5000) and ECL detection, as previously described [27]. Soluble protein was quantified using a Bradford assay with BSA as the standard.

### 2.4. RNA Sampling and Extraction

Cultures were harvested at the growth temperature either by vacuum filtration or by directly adding an aliquot to an equal volume of 5% phenol in EtOH. Cells in 5% phenol in EtOH were pelleted by centrifugation at 4 °C for 15 min (6000× *g*). Cell pellets were either frozen at −80 °C for up to three days or used immediately for RNA extraction. Cells were washed once with 50:100 TE and pellets suspended in lysis buffer (0.5% Triton × X-100, 0.5% N-lauroyl sarcosine, and 0.4% SDS in 50:100 TE). RNA was extracted via glass bead lysis in the presence of phenol followed by phenol-chloroform extraction and overnight precipitation with 4 M lithium chloride as described in Owttrim [32]. Following precipitation from lithium chloride, RNA was suspended in sterile water and precipitated at −80 °C with 0.10 volume NaOAc (3M, pH 5.2) and 2.5 volume ethanol (100%).

### 2.5. Quantification of RNA and Quality Confirmation

RNA concentration was determined using a NanoDrop spectrophotometer (Fischer Scientific, Edmonton, AB, Canada) and RNA quantity and quality was visualized by electrophoresis of denatured RNA (5 µg) on a MOPS-buffered 1.2% formaldehyde-agarose gel [32]. RNA quality was further confirmed using an Agilent 2100 Bioanalyzer (Agilent Technologies Inc., Waldbronn, Germany), as per the manufacturer’s protocol. A RIN number > 8.0 was required before proceeding with cDNA generation.

### 2.6. RNA Abundance

Northern analysis was performed as previously described [27]. Transcript size was estimated using Fermentas RiboRuler RNA markers.

### 2.7. cDNA Generation

Genomic DNA was removed from RNA samples (0.2 µg/µL) by treatment twice with DNase I (0.04 U/µL, Ambion) for 30 min at 37 °C. Use of DNase I treated RNA for standard PCR did not produce detectable amplification. cDNA was generated using DNase I treated RNA, random hexamers (Invitrogen, Waltham, MA, USA), and dNTPS (Thermo Fisher Scientific, Waltham, MA, USA) at 0.17 µg/µL, 50 ng/µL and 0.5 mM respectively. This mixture was hybridized in a MasterCycler Personal Thermal Cycler 5331 (Eppendorf) to 70 °C for 10 min, followed by 25 °C for 10 min, diluted in 5× First Strand Buffer (Invitrogen, Waltham, MA, USA) before addition of DTT (10 mM, Thermo Fisher Scientific, Waltham, MA, USA) and Superscript II (5 U/µL, Invitrogen). cDNA generation was performed using the following parameters: 25 °C for 10 min, 37 °C for 60 min, 42 °C for 60 min, 70 °C for 10 min.

### 2.8. Primer Design and Selection of Endogenous Control Gene

Three endogenous controls previously identified from microarray data were tested [33]. Primer pairs for *rnpB*, *rrn16sb* and *petB* were designed using Primer Express v3.0.1 software (Thermo Fisher Scientific, The Waltham, MA, USA) (Table S1). All three control genes gave constant abundance over various growth conditions and *rnpB* was selected as the endogenous control for ∆∆CT analysis. Primer annealing efficiency was determined to be >98% before further use.

### 2.9. qPCR Amplification

qPCR reactions were performed in triplicate in 96 well plates (MicroAmp Fast Optical 96-Well Reaction Plate with Barcode, Thermo Fisher Scientific, The Waltham, MA, USA). Each well contained 5 µL of 2× SYBR Mastermix (MBSU, University of Alberta), gene-specific forward and reverse primers (3.2 µM) and 2.5 µL diluted cDNA template (1:1024) in a final reaction volume of 10 µL. Amplification was performed by thermocycling at 95 °C for 2 min followed by 40 cycles of 95 °C for 15 s and 60 °C for 1 min on a 7500 Fast Real-Time PCR system (Thermo Fisher Scientific, The Waltham, MA, USA) at standard ramp speed. Melt curves were generated at 1 °C intervals from 60 °C to 95 °C.

### 2.10. Data Manipulation

Transcript abundance was normalized to *rnpB* in samples from differing conditions or strains and reported as ∆∆CT. Significance was determined using a Student’s *t*-test (*p* > 0.05). Raw data was collected from the 7500 Software V2.3 (Thermo Fisher Scientific, The Waltham, MA, USA) before statistical analysis and graphing performed in the GraphPad Prism 8 software version 8.0.1 (GraphPad Software, Inc., La Jolla, CA, USA).

## 3. Results

### 3.1. CrhR Protein Accumulation Is Regulated by Light Driven Reduction of the PQ Pool

**Figure 1:**
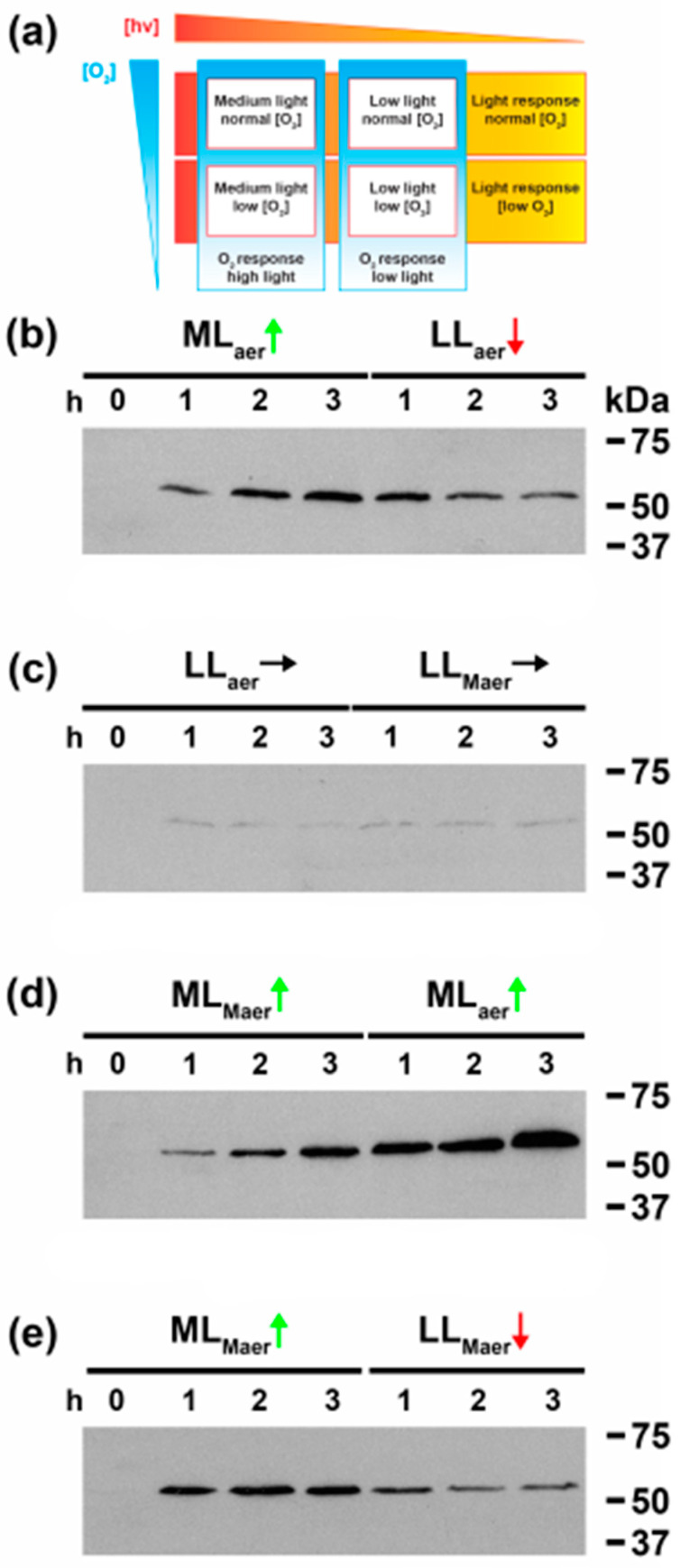
Combinatorial effects of varying oxygen availability and light quantity confirm redox regulation. CrhR accumulation was evaluated by Western blot analysis in wild type *Synechocystis* cells exposed to the indicated stresses. Cells were grown to mid-log phase at 30°C and subjected to: ML = Mid Light (150 µmol m^−2^ s^−1^); LL = Low Light (36 µmol m^−2^ s^−1^); AER = Aerobic conditions (bubbling with air); or MAER = Microaerobic conditions (bubbling with 95% N_2_ + 5% CO_2_). (**a**) Outline of interlinked experimental setup adapted from Barth et al. [12]. Experimental conditions are outlined in red. Response determined by transitioning between these conditions are displayed in red for light responses, and blue for O_2_ response. (**b**) Light response with air levels of O_2_. Cells grown under LL AER conditions were sampled every hour after transfer to ML for 3 h and upon return to LL for 3 h. (**c**) Light response with low O_2_. Cells grown under LL AER conditions were sampled for 3 h before moving to MAER for 3 h. (**d**) O_2_ response with ML. Cells grown under LL AER conditions were transferred to ML + MAER for 3 h before return to AER for 3 h. (**e**) O_2_ response with LL. Cells grown under LL AER conditions were transferred to ML + MAER for 3 h before return to LL for 3 h. Soluble protein (20 µg) was separated by SDS-PAGE and CrhR protein abundance determined by Western blotting using anti-CrhR antibody (1:5000) and ECL detection (26).

We have recently established that CrhR expression is controlled by a convergent sensing mechanism, activated by the common effect of abiotic stresses enhancing the redox poise of PQ in the ETC [11]. In order to differentiate redox expression from similar responsive mechanisms that regulate gene expression in photosynthetic organisms, experiments combining moderate light (ML) or low light (LL) under aerobic or microaerobic conditions were performed. The experimental design utilizes all combinations of low and high light and oxygen levels to generate eight growth conditions, as summarized in Figure 1a. As shown in Figure 1b, under aerobic conditions CrhR protein abundance was increased by ML and subsequently repressed by transfer to LL. In contrast, switching from aerobic to microaerobic conditions in LL had no effect on basal CrhR abundance (Figure 1c) while constant exposure to ML induced CrhR continuously under microaerobic conditions, an effect that was enhanced by a subsequent shift to aerobic conditions (Figure 1d). Finally, oxygen concentration did not affect the light response as ML induced while subsequent LL repressed CrhR abundance (Figure 1e). Overall, the results from the experiments shown in Figure 1 indicate that O_2_ concentration and thus reactive oxygen species (ROS) levels did not affect CrhR abundance whose expression was controlled in a reversible manner by the prevailing light quantity conditions. The resulting effect on CrhR accumulation induced by the 16 different combinations of altered ML, LL, microaerobic and aerobic conditions are summarized in Figure S1. It should be noted that in each case, CrhR abundance increased or decreased in growth conditions that reduced or oxidized the redox state of the ETC, respectively. CrhR abundance also altered in congruence with the presence or absence of light, however this was an indirect effect resulting from light alteration of the ETC redox poise and not a specific effect of light per se. It was also notable that ROS was not associated with CrhR expression, since oxygen tension did not affect accumulation. Together these results indicate that CrhR expression is regulated primarily by the redox poise of the ETC with a significant input from light and not by ROS or oxygen tension.

Since we have previously reported that CrhR associates with the thylakoid membrane under cold stress [34], we investigated the partitioning of CrhR into soluble and membrane fractions using different lysis techniques. As shown in Figure S2, CrhR was detected in both fractions after sonication or French Press lysis but solely in the soluble fraction after glass bead lysis. We therefore utilized this technique to ensure we isolated all CrhR polypeptide from each growth condition investigated.

**Figure 2:**
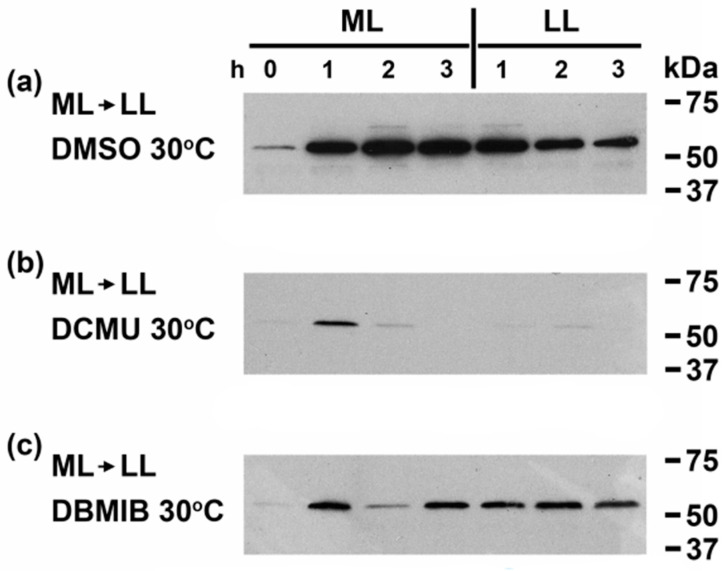
Light quantity and ETC inhibitor effect on CrhR accumulation at 30 °C. Wild type *Synechocystis* cells were grown to mid-log phase at 30 °C (T = 0) and the combined effect of light quantity and ETC inhibitors on CrhR accumulation was analyzed by Western blotting. (**a**–**c**) Light quantity. Cells grown at LL (36 µmol m^−2^ s^−1^) were sampled every hour after transfer to ML (150 µmol m^−2^ s^−1^) for 3 h and upon return to LL up to 3 h in the presence of DMSO (**a**); DCMU (**b**); DBMIB (**c**). CrhR protein abundance was determined by Western blotting, as described in Figure 1.

While we have previously shown that *crhR* expression is regulated by a number of abiotic stresses through their common effect on the redox poise of the ETC [10,11], we wished to further confirm that induction of CrhR protein accumulation was mediated by redox regulation under the growth conditions employed in Figure 1, the ETC inhibitors DCMU and DBMIB were used. The effect of ML on CrhR expression was initially determined at 30 °C in the absence of temperature stress (Figure 2a). Continuous ML exposure caused a rapid increase in CrhR abundance, a level that remained constantly elevated for the duration of exposure (Figure 2a). Culture shift from ML to LL reduced CrhR levels, indicating that ML induction was reversible and thus the factor causing CrhR induction (Figure 2a). Association of the redox poise of the ETC by ML was provided by DCMU substantially inhibiting ML induction compared to the control (Figure 2a), resulting from oxidation of the ETC downstream of Q_A_ at the inhibitor concentration used here (Figure 2b). In contrast, enhanced reduction of the ETC by DBMIB upstream of cyt *b*_6_*f* did not affect the ML induction but reversed the anticipated subsequent decrease of CrhR in LL (Figure 2c). This indicated that DBMIB enhancement of PQ reduction counteracted the decrease in electron flow through the ETC anticipated in LL.

### 3.2. Light Quantity and Light-Dark Transition Regulates CrhR Expression

**Figure 3:**
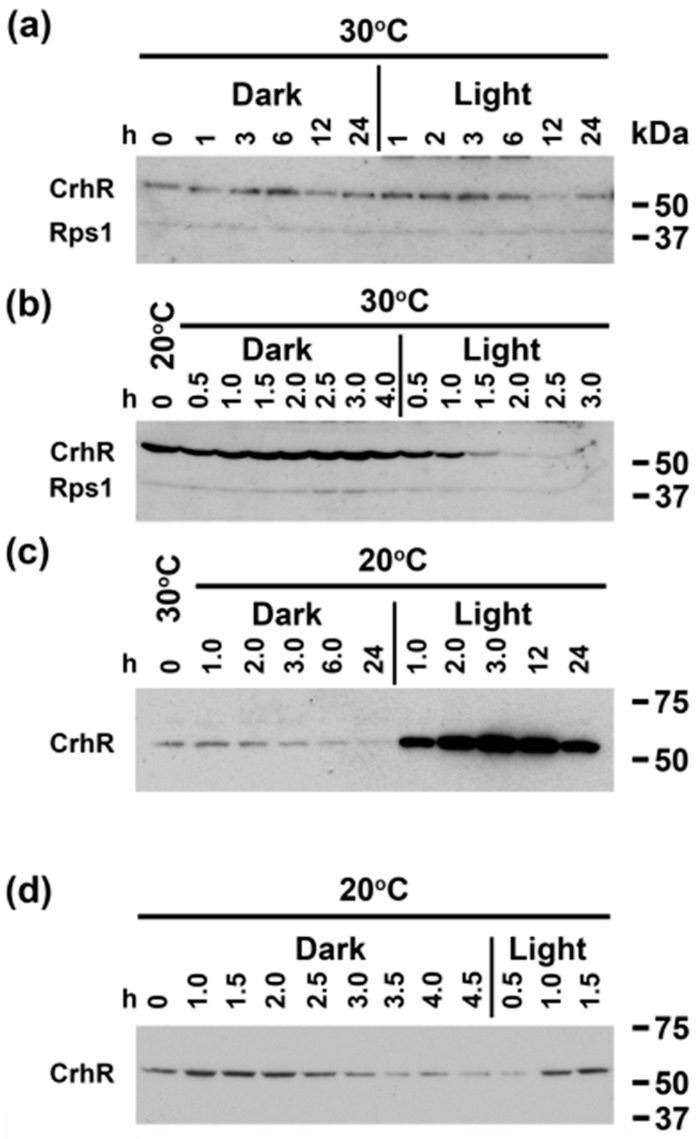
Effect of temperature shift and light-dark transition on CrhR accumulation. A *Synechocystis* culture was grown to mid-log phase under standard light conditions at 30 °C and aliquots subjected to the following treatments before determination of CrhR accumulation by Western blot analysis. (**a**) Cells were transferred to dark conditions at 30 °C for 24 h and subsequently exposed to light at 30 °C for 24 h. Cells were sampled at indicated times. (**b**) Cells were pre-induced for maximal CrhR accumulation for 3 h at 20 °C (T = 0) and then transferred to the dark at 30 °C for 4.5 h. The culture was then exposed to light for 3 h at 30 °C. Cells were sampled at indicated times. For (**a**,**b**), Western blots were simultaneously probed with antibodies against CrhR (55 kDa) and *E. coli* ribosomal protein S1 (Rps1, 40 kDa) which was used as a control for protein loading. (**c**) Cells were shifted simultaneously to the dark and 20 °C for 24 h and then exposed to light at 20 °C for 24 h. Cells were sampled at indicated times. (**d**) Cells were pre-induced for maximal CrhR accumulation for 3 h at 20 °C (T = 0) and then transferred to the dark at 20 °C for 4.5 h. Cells were subsequently transferred to the light at 20 °C for 1.5 h. Cells were sampled at indicated times. CrhR protein abundance was determined by Western blotting, as described in Figure 1.

Light quantity enhancement of CrhR protein abundance prompted further investigation of dark-light transition in conjunction with the presence (20 °C) or absence (30 °C) of temperature stress (Figure 3). Overexposure of the Western blot indicated that shifting *Synechocystis* from light to dark to light conditions at 30 °C had no substantial effect on the basal level of CrhR that is routinely observed (Figure 3a). However, in cultures pre-induced to maximum CrhR abundance by incubation at 20 °C for 3 h (Figure 3b 0 time), the expected repression of CrhR abundance produced by a temperature upshift to 30 °C was not observed in the absence of light (Figure 3b Dark). Subsequent exposure to light at 30 °C initiated the expected decrease in CrhR abundance (Figure 3b Light), indicating the requirement for a light signal to initiate CrhR degradation. Repeating the dark-light transitions at 20 °C in the absence (Figure 3c) or presence (Figure 3d) of CrhR pre-induction produced similar conclusions regarding the importance of a light signal to initiate both CrhR accumulation and degradation. In the absence of pre-induction, the expected low temperature induction of CrhR was not observed. Instead, the basal level of CrhR detected at 30 °C decreased extensively in the dark at 20 °C (Figure 3c Dark). Subsequent transfer to the light at 20 °C initiated the expected low temperature induction of CrhR, which remained elevated for the duration of low temperature stress (24 h) (Figure 3c Light). Similar results to those shown in Figure 3c Dark were also observed in cultures pre-induced to maximum CrhR induction at 20 °C, conditions that also caused a steadily decline in CrhR abundance in the dark at 30 °C (Figure 3d Dark). Again, transfer to the light at 20 °C resulted in CrhR induction (Figure 3d Light), similar to the observations shown in Figure 3c Light. Overall, the results suggest that a light signal is required for both low temperature induction and temperature upshift repression of CrhR accumulation.

### 3.3. Linking Light Quantity and PQ Redox Poise to Regulation of CrhR Expression

**Figure 4:**
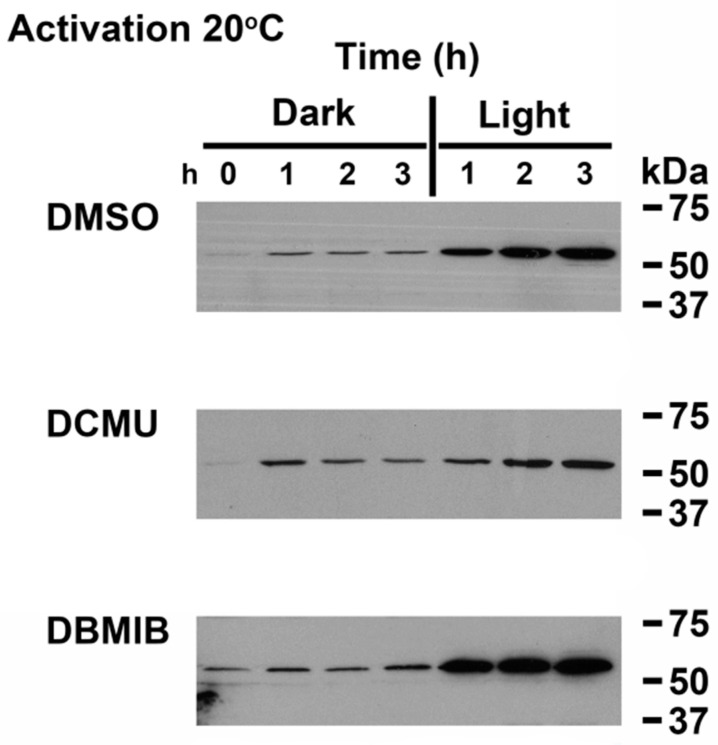
Absence of ETC reduction generates the dark effects. Wild type *Synechocystis* cells were grown to mid-log phase at 30 °C and the effect of dark-light transition associated with stresses that alter the ETC redox potential on CrhR accumulation was analyzed by Western blotting. Wild type *Synechocystis* cells grown at 30 °C under standard light conditions (T = 0) were transferred to 20 °C in the dark for 3 h after addition of DMSO, DCMU or DBMIB. Cells were then returned to growth light for 3 h. Cells were sampled at indicated times. CrhR protein abundance was determined by Western blotting, as described in Figure 1.

The results presented above indicate that dark-light transition had a dramatic effect on induction and repression of CrhR protein accumulation. This analysis was extended to include temperature shift in combination with ETC inhibitors and dark-light transition (Figure 4). To analyze low temperature induction, cells were grown at 30 °C (Figure 4 0 time) followed by concurrent transfer to the dark and 20 °C in the presence or absence of ETC inhibitors. Temperature has been previously demonstrated to affect expression of CrhR via effects on the redox poise of the ETC in the vicinity of PQ [11]. Treatment with the ETC inhibitors DCMU, which oxidizes PQ, and DBMIB, which reduces PQ, had divergent effects on CrhR expression, with the former preventing induction and the latter having no effect. As shown in Figure 4 DMSO, darkness again inhibited 20 °C induction in wild type cells, an effect that was reversed by transfer to the light. Low temperature induction of CrhR was also not observed in the dark in the presence of either of the ETC inhibitors, DCMU or DBMIB, however DCMU substantially inhibited (Figure 4 DCMU Light) while DBMIB enhanced the subsequent low temperature induction in the light (Figure 4 DBMIB Light). In this experiment, we demonstrated that the cells were still viable and thus used a sub-inhibitory concentration of DCMU. Commonly used DCMU concentrations (20 µM) completely abolish CrhR accumulation [10]. The DBMIB effect is indicative of restricted electron flow to the cyt *b*_6_/*f* complex thus enhancing reduction of PQ and increasing CrhR accumulation, as shown previously [10,11].

### 3.4. Light Quality Regulates CrhR Protein Expression at the Translational Level

**Figure 5:**
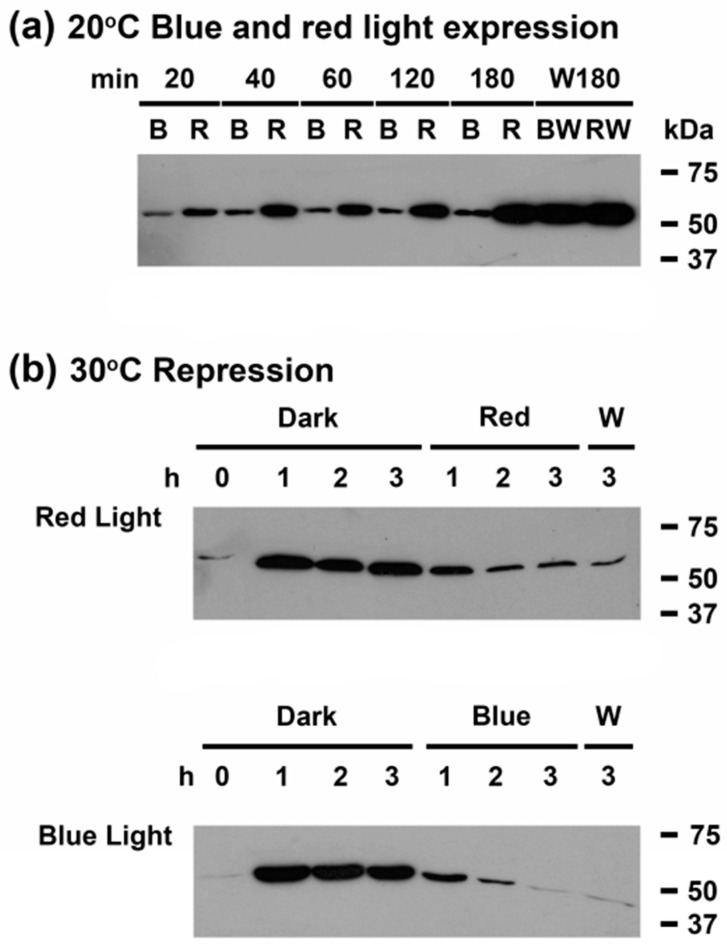
Red light is required for CrhR protein accumulation. Wild type *Synechocystis* cells were grown to mid-log phase at 30 °C and the effect of varying light quality on CrhR induction and proteolysis was analyzed in conjunction with temperature downshift and upshift by Western blotting. (**a**) Effect of light quality on low temperature induction. Cells grown at 30 °C under white light conditions were transferred to 20 °C in either blue (B) or red (R) light and sampled for 3 h at indicated time points. Cells were subsequently transferred to white light for 3 h (W180; BW, RW) to test for maximal CrhR accumulation. Protein derived from blue (B)- or red (R)-light treated cultures are displayed in adjacent lanes to aid visualization of the effects. (**b**) Effect of light quality on temperature upshift induced repression. Cells were grown at 30 °C under white light conditions (T = 0) before pre-induction at 20 °C for 3 h to induce maximal CrhR accumulation. Cells were subsequently returned to 30 °C in the dark and sampled every hour for 3 h. Cultures were then transferred to either red or blue light at 30 °C and sampled every hour for 3 h. Finally, the cultures were maintained at 30 °C and transferred to white light (W) to test for maximum CrhR repression (W3). CrhR protein abundance was determined by Western blotting, as described in Figure 1.

In order to further investigate the nature of the light signal required for low temperature induction of CrhR protein expression and the previously established redox regulation mechanism, the effect of varying light quality on temperature induction was determined. Cells were incubated in the dark at 30 °C for three h before exposure to either red (662 nm) or blue light (455 nm) at 20 °C. Induction of CrhR occurred normally in the presence of red light (Figure 5a R); both the rate of induction and the maximum level of CrhR reached occurred to similar levels as observed in white light (Figure 5a RW). This was not the case in the presence of blue light, where the expected cold induction was not observed (Figure 5a B). Induction was, however, subsequently restored by exposure of blue-treated cells with white light (Figure 5a BW).

### 3.5. A light Signal Is Required to Repress CrhR Expression

Repression of CrhR protein accumulation by proteolysis, which occurs upon return of cold shocked cells to the normal growth temperature of 30 °C, was also shown to require a light signal for induction (Figure 5b). In order to determine whether a specific wavelength of light was required to initiate proteolysis, concurrent treatment with red or blue light and temperature upshift after pre-induction of CrhR at 20°C for 3 h was performed. While temperature upshift induced proteolysis of CrhR was light-quality independent, proceeding to basal levels in the presence of both red and blue light (Figure 5b).

### 3.6. Far-Red Light Suppresses Red Light Induction of CrhR Accumulation

**Figure 6:**
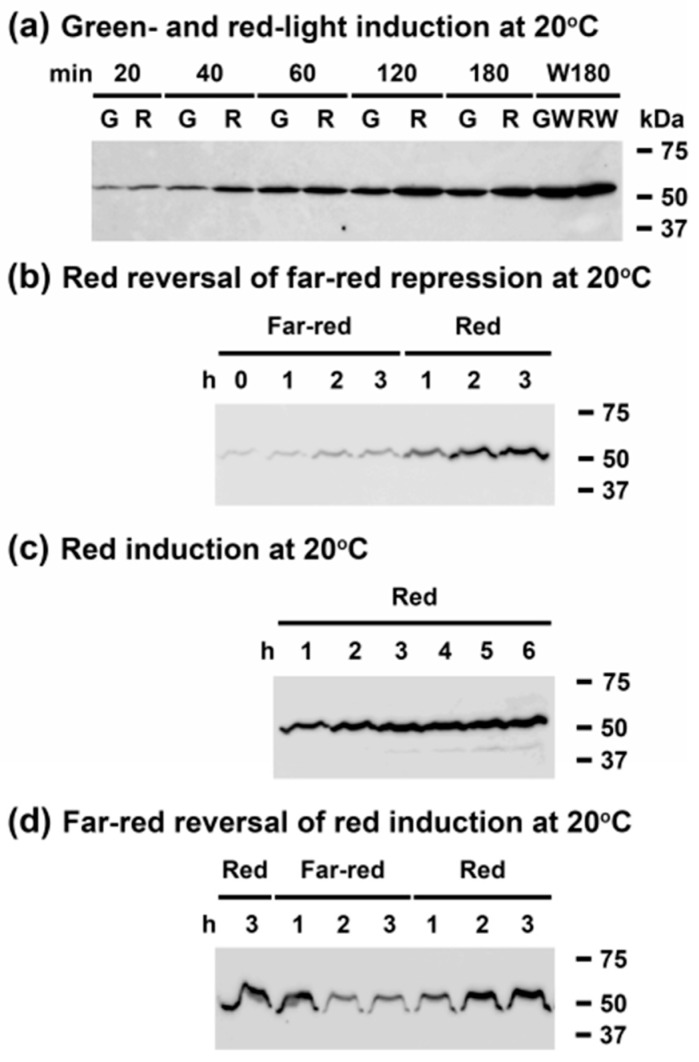
Far-red light reverses red light-induced CrhR protein accumulation. Wild type *Synechocystis* cells were grown to mid-log phase at 30 °C and the effect of varying green, red and far-red light quality on CrhR induction was analyzed in conjunction with temperature downshift by Western blotting. (**a**) Effect of green and red light on low temperature induction. *Synechocystis* cells grown at 30 °C under white light conditions were transferred to 20 °C in either green (G) or red (R) light and sampled for 3 h at indicated time points. Cells were subsequently transferred to white light for 3 h (W180; GW, RW) to achieve maximal CrhR accumulation. Protein derived from green (G)- or red (R)-light treated cultures are displayed in adjacent lanes to aid visualization of the effects. (**b**) Red reversal of far-red repression. *Synechocystis* cells grown at 30 °C under white light conditions (T = 0) were transferred to 20 °C in far-red light and sampled for 3 h at indicated time points (Far-red 1, 2, 3). Cells were subsequently transferred to red light for 3 h (Red 1, 2, 3). (**c**) Effect of red light on low temperature induction. As a control for red light induction kinetics, *Synechocystis* cells grown at 30 °C under white light conditions were transferred to 20 °C in red light and sampled for 6 h (Red 1, 2, 3, 4, 5, 6). Results shown in panels B and C were performed concurrently and subjected to Western blotting on the same blot and thus, the T0 time point shown in panel B also corresponds to T0 for panel C. (**d**) Far-red reversal of red induction. *Synechocystis* cells grown at 30 °C under white light conditions were transferred to 20 °C in red light for 3 h to induce maximum CrhR accumulation (Red 3). Cells were subsequently transferred to far-red light for 3 h (Far-red 1, 2, 3) and then returned to red light for 3 h (Red 1, 2, 3).

Since the blue- and red-light regulation of CrhR accumulation could be explained by the relative effect on the ETC redox poise or a photoreceptor-mediated event, it was of interest to expand analysis of the observed differential light quality regulation of CrhR accumulation. As shown in Figure 6a, green light induced CrhR accumulation at 20 °C to the same extent and with the similar kinetics as red light. In sharp contrast, CrhR induction was minimal in far-red light at low temperature (Figure 6b Far-red), an effect that was rapidly reversed by exposure to red light (Figure 6b Red). The rapid kinetics of red light induction to a maximal level at 20°C is shown in Figure 6c Red. Far-red reversal of red light induction at 20 °C was observed when cells induced in red light for 3 h (Figure 6d Red) were transferred to far-red light (Figure 6d Far-red). The observed far-red repression in these cells was rapidly reversed by subsequent exposure to red light (Figure 6d Red).

### 3.7. Light Quality Regulation of crhR Transcript Accumulation

**Figure 7:**
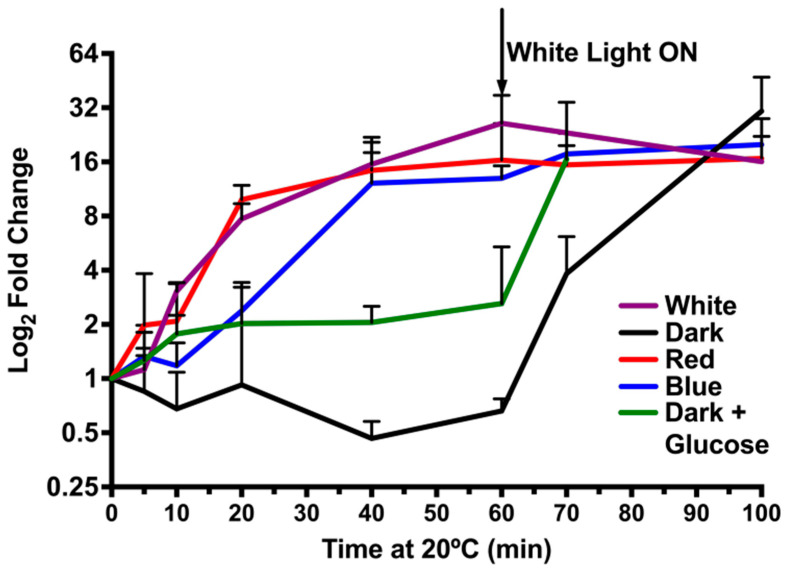
Reduction of the ETC is required for *crhR* transcript accumulation. Wild type *Synechocystis* cells were grown to mid-log phase at 30 °C and the effect of varying light quality on *crhR* transcript accumulation was analyzed in conjunction with temperature downshift. Aliquots were transferred to 20 °C at T = 0 in the absence (black line) or presence of glucose (green line) in the dark, or with continued illumination with white light (purple line), blue light (blue line) or red light (red line) for 60 min. At 60 min, all cultures were subsequently transferred to white light for an additional 40 min. *crhR* transcript levels were determined by qPCR and normalized to basal levels at 30 °C in white light. The data was obtained from two biological replicates. Error bars represent standard deviation.

Since there was a distinct difference in the ability of light quality to mediate temperature-responsive induction and repression of CrhR, similar analysis was performed at the transcript level. Enhanced accumulation of *crhR* transcript in response to temperature downshift in the presence of white light occurred much more rapidly than at the protein level (Figure 7). We observed a rate of *crhR* transcript accumulation in red light identical to that for white light and a decreased efficiency of induction by blue light (Figure 7). These effects are at least partially a reflection of the redox status of PQ, since red and blue light are known to result in a relative reduction and oxidation of PQ, respectively [13]. A light signal was required for *crhR* transcript accumulation as low temperature did not enhance *crhR* transcript abundance at 20 °C (Figure 7 Dark). While an intermediate level of transcript accumulation occurred in the presence of glucose in the dark, the levels were only significantly higher than observed in dark grown cells at the 40 min time point (Figure 7 Dark + Glc). Cells exposed to all conditions remained metabolically active, as transfer to white light at 20 °C induced transcript accumulation to similar levels (Figure 7 White Light ON).

### 3.8. crhR Translation Is Pre-Initiated at 30 °C but Elongation Is Inhibited

**Figure 8:**
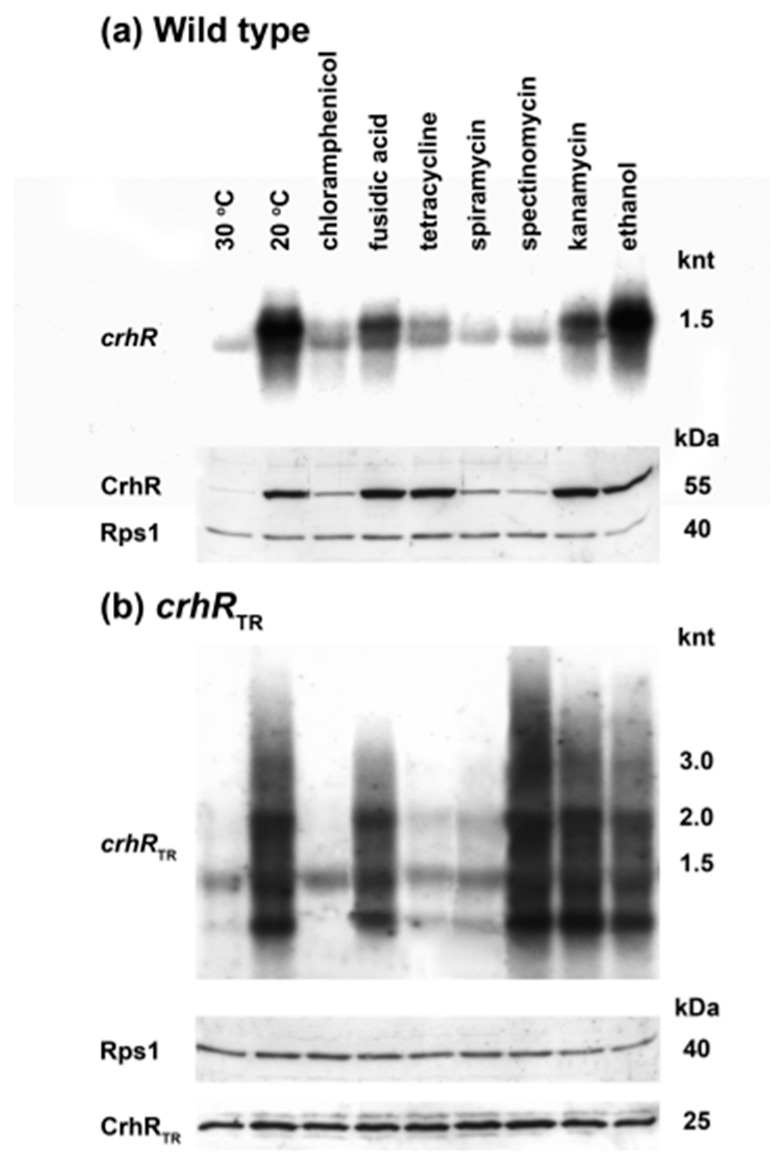
*crhR* translation is pre-initiated at 30 °C. (**a**) Wild type and (**b**) *crhR*_TR_ *Synechocystis* cells were grown to mid-log phase at 30 °C. The effects on *crhR* transcript and CrhR protein accumulation were evaluated in the presence of inhibitors of translation initiation (kanamycin, fusidic acid and tetracycline) and translation elongation (chloramphenicol, spiramycin and spectinomycin). Ethanol, used to dissolve the antibiotics, was included as a control. The indicated antibiotics (150 mg/mL) were added and incubation continued for an additional 1 h at 30 °C to fully inhibit de novo protein synthesis. Cultures were transferred to 20 °C and incubated for 1 h for maximum induction of *crhR* expression. Aliquots from these cultures were harvested for RNA and soluble protein extraction were processed for northern and western analyses as described previously [27,32]. *crhR* (~1500 nt) and the truncated *crhR*_TR_ (~750 nt) transcripts were detected from total RNA probed with a 93 bp *Hinc*II-*Sac*II internal fragment of *crhR*. Western blots using protein isolated from the cultures used for Northern analysis were simultaneously probed with antibodies against CrhR (55 kDa) and *E. coli* ribosomal protein S1 (Rps1, 40 kDa), used as a control for protein loading, and detected by ECL, as described in Figure 1.

The ability of light quality to induce cold-shock mediated expression of *crhR* transcript and protein suggests the involvement of a post-transcriptional or translational regulatory mechanism. Using inhibitors that differentially affect translational initiation or elongation, the requirement for de novo protein synthesis was assessed in cold-stressed wild type and cells lacking functional CrhR RNA helicase activity in the partial genomic deletion mutant, *crhR*_TR_. The expected basal levels of *crhR* transcript and protein at 30 °C are detected in wild type cells (Figure 8a 30 °C). This basal expression differs significantly from the enhanced transcript level observed upon temperature downshift to 20 °C in wild type cells (Figure 8a 20 °C). Transcript and protein accumulation was differentially affected depending on the mode of action of the translational inhibitor in wild type cells (Figure 8a Wild type). The presence of inhibitors of translation elongation (chloramphenicol, spiramycin and spectinomycin) did not allow low temperature induction of transcript or protein above basal levels observed in wild type cells at 30 °C (Figure 8a Wild type *crhR* and CrhR), while inhibition of translation initiation (kanamycin, fusidic acid and tetracycline) did not affect low temperature induction at either the transcript or protein level (Figure 8a *crhR* and CrhR). Ethanol, used to dissolve the antibiotics, was included as a control. Furthermore, it is of note that two *crhR* transcripts were differentially detected (Figure 8a *crhR*), with the faster migrating transcript present under all conditions and generated the basal level of CrhR protein (Figure 8a Wild type). From the perspective of the low temperature induction mechanism, it was insightful that the slower migrating transcript was observed to accumulate only under conditions that resulted in induction of CrhR protein abundance. Furthermore, CrhR protein accumulation only occurred to a maximum level irrespective of transcript abundance (Figure 8a Wild type). These results are associated with *crhR* being encoded in an operon, *rimO-crhR*, that is differentially expressed and processed in response to temperature shift [26].

In sharp contrast, the absence of functional CrhR RNA helicase activity dramatically altered the pattern of both transcript and protein accumulation in the partial deletion mutant, *crhR*_TR_ (Figure 8b *crhR*_TR_). The basal level of a single *crhR*_TR_ transcript was slightly enhanced above wild type levels at 30 °C while four stable transcripts accumulated in the partial *crhR*_TR_ deletion mutant in the presence of translation initiation inhibitors at 20 °C. These stabilized transcripts resulted from multiple *rimO-crhR* operon processing events, as previously described [26,27] (Figure 8b *crhR*_TR_). Again, as observed under conditions that induced CrhR in wild type cells (Figure 8b CrhR), CrhR_TR_ protein accumulated to a constant maximal level in the absence of functional CrhR helicase activity under all conditions tested, irrespective of transcript abundance, inhibitor presence or temperature (Figure 8b CrhR_TR_). This suggests that functional CrhR helicase activity is required for CrhR degradation and that the C-terminus contains a domain required for degradation.

The antibiotic results provide further confirmation that *crhR* transcript and protein expression is regulated post-transcriptionally, specifically by an inhibition of translation elongation at 30 °C. Importantly, this blockage is present at 30 °C, indicating that translation of the *crhR* transcript has pre-initiated at this temperature but elongation is halted. Light quality combined with translation elongation regulation suggests a role for phytochrome in the regulatory mechanism.

## 4. Discussion

**Figure 9:**
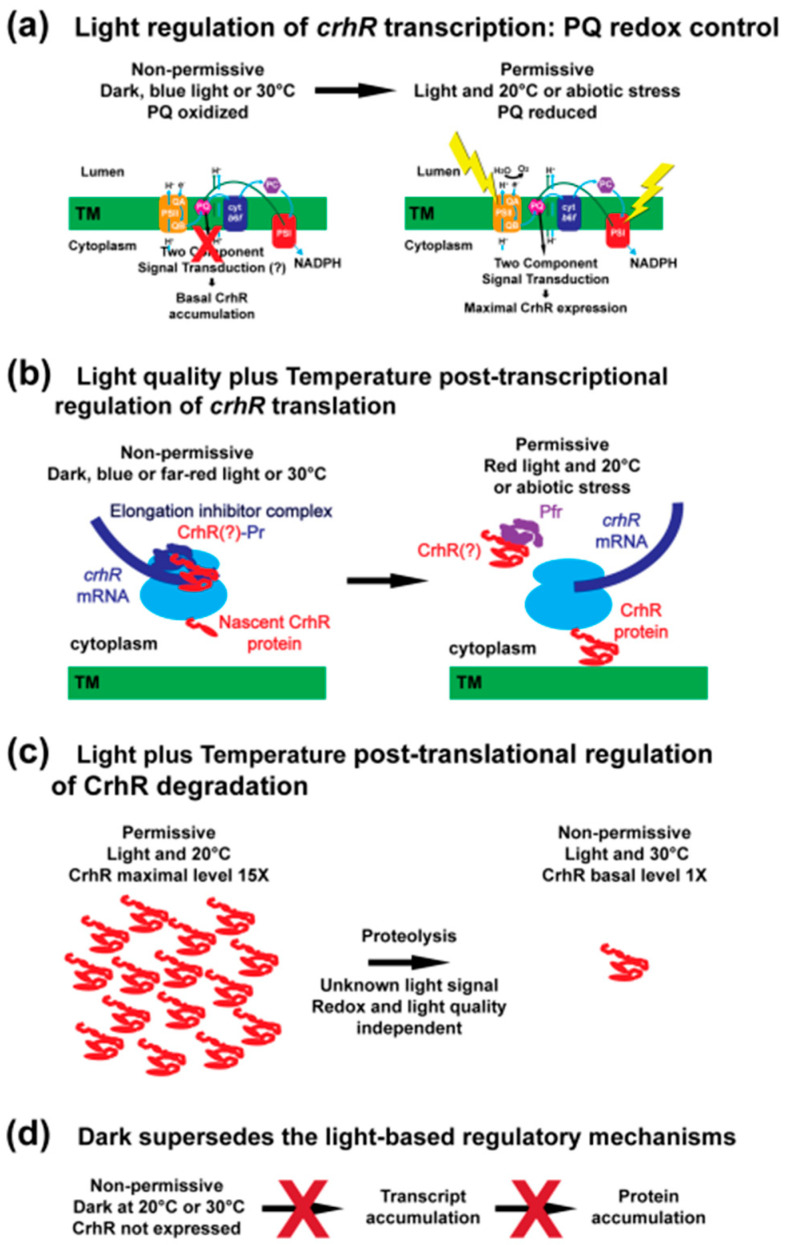
Summary: Discrete light signals regulate *crhR* expression. (**a**–**c**) Three discrete light signals regulate *crhR* expression at distinct points. (**a**) Light driven redox regulation. Under non-permissive conditions when CrhR is not required (dark, blue light, or 30 °C) the relatively oxidized PQ pool does not activate a two-component signal transduction chain leading to enhanced *crhR* transcript accumulation. This unknown signal transduction pathway is activated by permissive conditions (light and 20 °C or abiotic stress) yielding *crhR* expression. (**b**) Light quality regulation of CrhR accumulation. For conditions under which CrhR is not required and/or expressed (dark, blue light or 30 °C) we propose that translation has initiated on *crhR* transcripts however elongation has halted, potentially involving binding of Pr or an unknown factor, potentially CrhR (Crh?). Transfer to permissive conditions where CrhR is expressed or required (red light and 20 °C) relives the translational block allowing translation elongation and full-length CrhR production. In response to dark-light transfer, conversion of Pr-CrhR to Pfr-CrhR or binding of activated Pfr to CrhR could remove the translational block. (**c**) Light-dependent proteolysis. A redox- and light quality-independent light signal is required for induction of CrhR proteolysis in response to temperature upshift from 20 °C to the non-permissive condition, 30 °C. (**d**) Dark supersedes the light-based regulatory mechanisms. *crhR* transcript and protein are not observed to accumulate at 20 or 30 °C. This indicates that darkness counteracts the low-temperature and light induction signals, reinforcing the requirement for light signals for expression.

In order to survive, photoautotrophic organisms including cyanobacteria, depend on the ability to sense and respond to light, whose harvesting provides the energy required to form carbon skeletons via photosynthetic carbon fixation. To accommodate the diverse range of light regimes cyanobacteria experience in nature, they have evolved an expanded repertoire of light-sensing systems that modulate gene expression using diverse mechanisms. The importance of light sensing and signaling was exemplified by the diversity of light parameters that regulated *crhR* expression. Here, we show that three distinct light signals affected *crhR* expression at discrete points, as summarized in Figure 9. Fundamentally, a light- or exogenous glucose metabolism-derived enhancement of ETC reduction is required for *crhR* transcript accumulation, as shown here and previously [10,11,27] (Figure 9a). This is a crucial signal as if this redox signal is not perceived *crhR* is not expressed, as observed in dark treated cells. Subsequently, a light quality signal is required for CrhR protein accumulation thereby implying that red-light activation of a member of the *Synechocystis* phytochrome superfamily facilitates translation of the *crhR* transcript (Figure 9b). The third signal is also contingent on a light signal per se to repress CrhR protein accumulation by proteolysis in the absence of stress but is ETC redox poise- and light quality-independent (Figure 9c). The crucial role performed by light was emphasized by the observation that dark treatment inhibited all three light-regulated pathways (Figure 9d). Although the results presented here are in contrast to data suggesting that cold induction of *crhR* transcript accumulation occurred in the dark [17,31], they agree with data indicating that light was required for both *crhR* transcript and protein accumulation [10,30]. Overall, CrhR appears to play a role in maintaining homeostasis of photosynthesis in response to conditions resulting in over-reduction of the ETC. This crucial conclusion is supported by the results presented here combined with previous physiological and omics analysis, which suggest that CrhR regulates translation of protein products required to adapt to environmental conditions that perturb ETC homeostasis [11,25,27,35].

The light and dark signals are not all contingent on the redox poise of the ETC as only the primary signal required for expression is associated with light-quantity stimulated reduction of PQ that enhanced *crhR* transcript accumulation, as based on the following observations. While various analyses have indicated that the redox poise of the cyanobacterial ETC is relatively reduced in dark grown cells compared to land plants, subsequent light exposure significantly enhances this level [36,37,38]. Similarly, while light quality studies have shown differing reducing levels, all wavelengths reduce the ETC which would imply that *crhR* should be expressed under all light qualities [38,39]. This was not the case observed here. While red light induced *crhR* transcript accumulation, as potentially expected from enhanced ETC reduction, the transcript was not translated, indicating post-transcriptional regulation. In conjunction, red light induction was reversible by far-red light. The combined results indicating that a member of the extended *Synechocystis* phytochrome family is required for *crhR* transcript translation. With respect to the light signal required for the post-translational proteolysis of CrhR occurring upon stress removal, we show that neither redox- nor phytochrome- mediated mechanisms are involved. Finally, the observation that dark treatment overrides/subverts all three light signals is crucial since *crhR* is not expressed under these conditions even though the ETC is reduced to a minimal level. Thus overall, the results indicate there is a greater than zero threshold reduction level of the ETC that is required to induce *crhR* expression. We have previously shown this in response to exogenous glucose metabolism in dark treated cells in which *crhR* transcript and protein abundance are enhanced to a level intermediate between undetectable levels in the dark, even when the ETC is not fully oxidized and light- treated conditions with substantially enhanced ETC reduction [10].

### 4.1. The Role of Light-Mediated Alteration of the PQ Redox Poise

Utilization of an interlinked experimental system allowed us to assess the individual contributions of light, redox and ROS on gene expression [12]. These results confirmed and extended previous evidence [10,11] that the redox poise of the ETC was the primary driver of CrhR expression with light not unexpectedly playing an important role (Figure 9a). This analysis further demonstrated the interconnection between light and redox regulatory pathways as CrhR exhibited a protein expression pattern termed redox^II^, associated with ROS detoxification, redox regulation/balance and redox signaling in *C. reinhardtii* [12]. Light quantity regulation of CrhR expression was also associated with the redox state of PQ, as the ETC inhibitors DCMU and DBMIB that divergently affect PQ redox poise, decreased and did not alter CrhR accumulation in response to high light, respectively. The associated inhibitor and light-dark alteration of ETC redox poise has previously been confirmed by PAM analysis [36]. Together, these results indicated that activation of a two-component signal transduction pathway by reduced PQ, and not light per se, is required for *crhR* transcript accumulation, as shown in Figure 9a.

### 4.2. Light Quality Regulates crhR Translation

Here, light quality was also shown to be crucial for *crhR* expression as although transcript accumulated in both red and blue light in response to low temperature stress, CrhR protein only accumulated in red light. This implies that red-light activation of a member of the *Synechocystis* phytochrome superfamily [6] was required for *crhR* translation, but not transcription, in response to abiotic stress, as shown in (Figure 9b). Involvement of a phytochrome family member was supported by the observation that far-red light reversed red light induced CrhR accumulation, even under permissive conditions, growth at 20 °C. As expected for phytochrome regulation, red induction was repressed by far-red exposure, while red light subsequently induced CrhR expression. Interestingly, the kinetics of CrhR red-light induction and far-red repression were similar and they also matched induction and reversal by exposure to and removal of diverse abiotic stresses [11,27,28].

Identification of the family member involved in phytochrome-mediated CrhR regulation will require further analysis. Potential candidates would be the Cph1-Cph2 photoreceptors known to exhibit photoconversion by red and far-red light [40,41]. However, similar to our results, although *crhR* transcript was shown to be red light inducible and not affected by far red light, its abundance was not altered in *cph* single or double mutants [15]. This potentially implies that other phytochrome-related proteins in the *Synechocystis* genome are involved, as interpreted for other red-far-red responsive genes by Hübschmann et al. [15]. It is also interesting to note that although Hübschmann et al. [15] showed *crhR* transcript enhancement by red light, *rimO* (*slr0082*) abundance was not affected. *crhR-rimO* are encoded as an operon in the *Synechocystis* genome whose full length transcript is rapidly processed into monocistronic transcripts [26]. This implies that red light specifically affects *crhR* transcript accumulation at the post-transcriptional level, adding a further level of regulation to this operon.

In addition, CrhR induction in green light suggests the CcaS/CcaR two-component system is not associated with CrhR regulation since CcaS transitions in response to red-green and not red-far-red shifts [42]. Overall, the light-quality regulation by green, blue, red and far-red light indicates that CrhR expression is, in part, mediated by red to far-red phytochorome photoconversion.

Phytochrome is a major regulator of gene expression in both higher plants and cyanobacteria known to control key physiological processes by sensing a range of wavelengths [6,15,43,44,45]. In *Synechocystis*, we envisage a mechanism of CrhR regulation in which binding of a factor, potentially CrhR, to the *crhR* transcript under non-permissive conditions, dark, blue or far-red light or 30 °C, would inhibit translation elongation (Figure 9b). Relief of translation inhibition would require removal of the inhibiting protein by interaction with a red light-activated photoreceptor, allowing translation under permissive conditions, either cold stress or red light (Figure 9b). Evidence for a similar regulatory system has been presented in *Synechocystis* where translation elongation of the *psbA*-encoded D1 protein, a PSII component, is arrested by an unknown mechanism in the dark. An unspecified light signal then targets the ribosome-PsbA nascent chain complex to the thylakoid membrane where translation is completed [46]. Thus, expression of genes associated with crucial aspects of photosynthesis are regulated by a variety of light signals in cyanobacteria. However, regulation of a single gene by multiple light signals has, as far as we are aware, not been reported.

The proposed phytochrome-mediated translational regulation mechanism is shown in Figure 9b, was also supported by the observation that low temperature induction of *crhR* continued in the presence of antibiotics that inhibit translation initiation while, elongation inhibitors blocked *crhR* expression. This suggests that *crhR* translation was pre-initiated at the non-permissive condition, 30 °C, but elongation was stalled, similar to D1 regulation. We hypothesize stalling was mediated by CrhR binding, as corroborated by the observation that translational control was not observed in the absence of functional CrhR RNA helicase activity. Transfer to the permissive condition, 20 °C in combination with red light, could remove CrhR via interaction with activated Pfr thus alleviating the elongation inhibition and allowing translation to proceed. These results support the conclusion that a phytochrome-mediated mechanism regulates the post-transcriptional accumulation of CrhR protein via an auto-regulatory process (Figure 9b). This conclusion was supported by the observation that the *crhR* transcript is a major target for CrhR binding [35]. Together, the data suggest that a potentially conserved phytochrome-mediated mechanism imparts translational control in response to light signals in cyanobacteria, with CrhR performing an active role in the process.

### 4.3. A Light Signal Regulates CrhR Proteolysis

Repression of light-dependent responses is a crucial requirement to maintain cellular fitness in plants and cyanobacteria. Examples include regulation of stomatal development and phytochrome responses in plants [47,48] and D1 and phycobilisome turnover in cyanobacteria [49,50]. CrhR abundance is also regulated at the post-translational level, as abiotic stress enhanced CrhR expression is alleviated by conditional proteolysis in response to upshift to non-permissive temperatures and removal of a divergent range of stresses at 30 °C [11,28]. Three pieces of data presented here confirm and expand on these observations, indicating that induction of CrhR proteolysis required an unspecified light signal: (1) CrhR repression did not occur in dark treated cells; (2) CrhR proteolysis also proceeded independently of redox regulation, since degradation occurred normally in the presence of various ETC inhibitors as shown by Ritter et al. [11]; and (3) the proteolytic repression of CrhR accumulation in response to temperature upshift was not light quality dependent, since CrhR degradation was observed under all wavelengths tested, as diagramed in Figure 9c.

### 4.4. Dark Incubation Supersedes All Three Light Signal Responses

Finally, the overall importance of light signals for *crhR* expression was provided by the observation that dark conditions superseded both the low temperature and all three light induction signals (Figure 9d). These observations emphasize the requirement for CrhR catalyzed RNA helicase activity in growth conditions that reduce the ETC and promote photosynthesis. The dark-light regulation of *crhR* expression also implies involvement with functioning of the circadian clock [51,52].

## 5. Conclusions

In summary, we provide insights into the physiology and molecular biology involved in *Synechocystis* sensing the external environment. We show that a complex series of discrete light sensing and signaling events contribute to the regulation of *crhR* expression at distinct levels. These findings are unique, as while gene regulation by various light parameters is well characterized, sensing of divergent light signals that function at the transcript accumulation, translation and post-translational levels to regulate a single gene has not been reported. These mechanisms, especially the pre-initiation of *crhR* translation under non-permissive conditions, would allow for rapid adjustment of CrhR abundance, aiding acclimation to the range of light and abiotic stress conditions *Synechocystis* continuously encounters in the natural habitat. The mechanisms by which the identified light and redox signals are sensed and transduced to produce an integrated response altering CrhR expression and the concomitant downstream alteration in gene expression required for stress acclimation remain outstanding questions that are addressed in Ritter et al. [53].

## Data Availability

Data are contained within the article and Supplemental Files.

## Supplementary Materials

The following supporting information can be downloaded at: https://www.mdpi.com/article/10.3390/cells11213397/s1, Table S1: Primers used in this study; Figure S1: Summary of oxygen concentration and light quantity effect on CrhR expression; Figure S2 CrhR partitioning into soluble and membrane fractions.
