# Supplementary material for: Multiple Light-Dark Signals Regulate Expression of the DEAD-Box RNA Helicase CrhR in Synechocystis PCC 6803
Source: Cells. 2022 Oct 27;11(21):3397. doi: 10.3390/cells11213397 (PMC9655292; doi:10.3390/cells11213397)
Supplement: Supplementary file 1 [file cells-11-03397-s001.zip › cells-1978406-supplementary.pdf]

Supplementary Material

Multiple light-dark signals regulate expression of the DEAD-box RNA helicase CrhR in *Synechocystis* PCC 6803

Sean P.A. Ritter[1], Logan A. Brand[2], Shelby L. Vincent[2], Albert Remus R. Rosana[2], Allison C. Lewis[3], Denise S. Whitford[2] and George W. Owttrim[2]*

[1]Department of Botany, University of British Columbia, 3156-6270 University Blvd., Vancouver, BC Canada V6T 1Z4

[2]Department of Biological Sciences, University of Alberta, Edmonton, AB, Canada T6G 2E9

[3]Max Planck Institute of Molecular Cell Biology and Genetics, Pfotenhauerstr. 108, 01307 Dresden, Germany

Corresponding author

*G. W. Owttrim, Department of Biological Sciences, University of Alberta, Edmonton, AB, Canada T6G 2E9. Telephone, +1-780-492-1803, Fax, +1-780-492-9234, E-mail, gowttrim@ualberta.ca; ORCID ID 0000-0002-4709-2091

File contains: **Table S1**

Figure S1 and Figure S2

Table S1: Primers used in this study

crhR Forward Primer	GATCGCCGCTGCTGCTT
crhR Reverse Primer	GGCACTTCCCAATCGGATT
rnpB Forward Primer	TGTCACAGGGAATCTGAGGAAAGT
rnpB Reverse Primer	CTGTTTACTGGTTGCTGTTTTCTAAAA
petB Forward Primer	TGGGCGGTAAAAATCGTTTC
petB Reverse Primer	TCGCATGAGGGTCACCAATT
rrn16sb Forward Primer	ATCAAACCCGGCCTCAGTTC
rrn16sb Reverse Primer	ACCTGCGATTACTAGCGATTCC

	**Box I (High Light + O2 -> Low Light +O2)**	**Box II (Low Light + O2 -> Low Light + Anoxia)**	**Box III (High Light + Anoxia -> High Light + O2)**	**Box IV (High Light + Anoxia -> Low Light + Anoxia)**
**Tested Effect**	**Light Effect + ROS**	**LL Effect +/- ROS**	**HL Effect -/+ ROS**	**Light Effect no ROS**
**Observed CrhR Expression Pattern**	⇑⇓	⇒⇒	⇑⇑	⇑⇓
**Light Regulation**	⇑⇓	⇒⇒	⇑⇒	⇑⇓
**ROS Regulation**	⇑⇓	⇒⇓	⇒⇑	⇒⇒
**Redox Regulation**	⇑⇓	⇒⇒	⇑⇑	⇑⇓
**Light Repressed Regulation**	⇓⇑	⇒⇒	⇓⇒	⇓⇑
**"4-Down" Regulation**	⇑⇓	⇒⇒	⇓⇑	⇓⇑

Figure S1 Summary of oxygen concentration and light quality effects on CrhR expression. A summary of the effects of manipulating oxygen concentration and light intensity on CrhR accumulation from data shown in Figure 1 is provided. The five patterns of protein accumulation observed in *C. reinharditii* are listed on the left (Barth et al., 2014). The experimentally observed pattern of CrhR accumulation is shown with black arrows. CrhR accumulation patterns that match those observed in *C. reinharditii* are depicted as green arrows while results that do not match are shown as red arrows. Vertical, horizontal and down arrows indicate increased, no change and decreased CrhR accumulation, respectively.

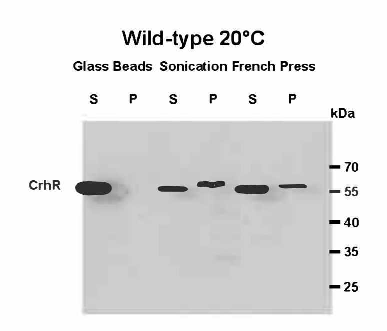

Figure S2 CrhR partitioning into soluble and membrane fractions. Wild type *Synechocystis* cells were grown to mid-log phase at 30°C and cold induced for maximal CrhR accumulation at 20°C for 3 h. Cells were lysed by vortexing in the presence of glass beads for 15 cycles of ec each, sonication for 15 cycles of ec each or passage through a continuous flow French press three times. Soluble and insoluble, membrane containing fractions, were obtained by centrifugation at 10,000 x g for 10 min. For soluble fractions µg of protein was used for Western analysis. The insoluble pellet was dissolved in the same volume as recovered in the soluble extract and a volume equal to that used from the soluble extract was subjected to Western analysis. Western blots were probed with antibodies against CrhR (55 kDa) and detected by ECL.
